# Acute One-Cigarette Smoking Decreases Ghrelin Hormone in Saliva: A Pilot Study

**DOI:** 10.1155/2014/575671

**Published:** 2014-04-07

**Authors:** Yahia A. Kaabi, Mohiealdeen A. Khalifa

**Affiliations:** ^1^College of Applied Medical Sciences, University of Dammam, Al-Khobar 31441, Saudi Arabia; ^2^College of Medical Rehabilitation Sciences, Taibah University, Al-Madinah Al-Munawwarah 21423, Saudi Arabia

## Abstract

Cigarette smoking is commonly associated with weight loss and mechanisms for these weight changes are still elusive. Ghrelin is a peptide hormone that works in a neuroendocrine fashion to stimulate hunger and the desire for food intake. Ghrelin is also secreted in saliva, probably to enhance food taste. In the current study, we tested the direct impact of acute cigarette smoking on total ghrelin found in saliva. *Methods*. Blood and saliva samples were collected from 30 healthy nonsmoker male volunteers before and after one-cigarette smoke. Total ghrelin in serum and saliva was measured by ELISA based method. *Results*. Data showed a statistically significant reduction in salivary ghrelin after smoking (*P* < 0.0001). In serum, total ghrelin levels were not affected before and after smoking (*P* = 0.1362). Additionally, positive correlation was observed between serum and salivary ghrelin before smoking *(r* = 0.4143 and *P* = 0.0158); however, this correlation was lost after smoking (*r* = 0.1147 and *P* = 0.5461). *Conclusion*. Acute one-cigarette smoking can negatively affect ghrelin levels in saliva that might contribute to the dull food taste in smokers.

## 1. Introduction

Cigarette smoking is common and one of the major health problems around the globe. Chronic smokers often suffer from loss of appetite, abnormal food taste, and weight loss [1, 2]. Possible mechanisms for these conditions are related to both metabolic effects of tobacco smoking and neurologic effects of nicotine, the main addictive component found in smoke, on appetite [3]. Cigarette smoking has shown to increase the metabolic rate and energy expenditure and reduce caloric intake [4, 5]. All can result in a negative energy balance and finally weight loss. Exposure to cigarette smoke has also been reported to cause loss of appetite via central hypothalamic mechanisms in mice [6, 7]. Additionally, nicotine treatment in humans has also been shown to cause anorexic effects and food restrictions while positively correlating with feeling of fullness [8, 9]. On the other hand, smoking cessation causes fat deposition and weight gain [10–12]. Although it is clear therefore that cigarette smoking can manipulate body weight, however, the exact mechanisms are still unclear.

The appetite hormone ghrelin is a 28-amino acid acylated polypeptide secreted from the oxyntic cells of the stomach wall. It has been recognized as the natural ligand to an orphan G-protein-coupled receptor called growth hormone secretagogue receptor (GHS-R) [13]. Ghrelin administration induces appetite, food intake, and weight gain in humans [14, 15]. Apart from systemic secretion, ghrelin is found to be produced and released by cells of the parotid and submandibular salivary glands [16, 17] as well as taste bud cells [18]. The exact function of ghrelin present in saliva requires further investigations; however, this salivary secretion suggests a direct modulatory role of ghrelin on taste bud cells signaling and function that might lead to the enhancement of food taste and appetite for food ingestion [18].

In this study we investigated the direct effect of acute cigarette smoking on the ghrelin hormone found in saliva in order to establish an additional link between cigarette smoking and disrupted food taste and loss of appetite.

## 2. Subjects and Methods

### 2.1. Subjects

30 male student volunteer subjects (age range 18–25 years) participated in this study. All participants were asked to sign a consent form for a written local ethical approval and checked for pulse rate, temperature, blood pressure, body mass index (BMI), and glucose level. A questionnaire was designed to exclude subjects with either of the following exclusion criteria: obesity (BMI ≥ 30), history of gastric surgery, eating disorders, and diabetes mellitus.

### 2.2. Samples Collection

Fasting blood and saliva samples were initially collected from all 30 subjects. Then they were asked to smoke one filtered cigarette containing 0.9 mg of nicotine for five minutes with a puff lasting for at least 3 seconds every 15-second time intervals. 10 minutes later other blood and saliva samples were obtained. Venous blood was collected under aseptic phlebotomy technique in plain tubes and serum supernatant was recovered and frozen at −80°C until the assay was performed. For saliva, the Versi.SAL1 saliva collection device was used (Oasis diagnostics corporation, WA, USA). All participants were instructed to rinse their mouth thoroughly with tap water, place the spongy part of the Versi.SAL1 directly under the tongue, and stimulate saliva secretion by binding the neck until the sponge is filled. Saliva was then transferred into clean prechilled 1.5 Eppendorf tubes and centrifuged for 10 minutes at 4,000 ×g and 4°C to remove cellular debris. The supernatant was then recovered in 1.5 Eppendorf tubes and stored at −80°C until the assay was performed.

### 2.3. Ghrelin Assay

Total ghrelin in all subjects' samples (serum and saliva) was measured using the human ghrelin TSZ-ELISA 96 well-plate kit (TSZ-ELISA, MA, USA). The assay was performed according to the manufacturer's procedure. Briefly, each sample was diluted 10x in the sample diluent and 50 *μ*L was transferred into the corresponding wells in triplicate. The plate was incubated for 30 minutes at 37°C followed by addition of HRP enzyme labeled antibody for 30 minutes. Chromogen/substrate solution was added to each well for color development. The reaction was stopped and the optical densities (OD_450_ nm) for standards sand samples were determined. Standards' absorbance values were subtracted from the average value of the zero standards and plotted graphically against their corresponding concentrations using linear regression curve-fit generated by GraphPad Prism software (San Diego, California, USA). The unknown concentrations were determined by direct interpolation using the same software.

### 2.4. Statistical Analysis

Data were first checked for normality to satisfy the assumption of normal distribution. Because the data were derived from the same subjects before and after smoking, they in fact self-control each other to eliminate variation within subjects. Means and standard error of the mean (SEM) were calculated for each data set. Then a paired student's *t*-test was carried out to compare between means at 95% confidence intervals. For correlation analysis a Pearson's correlation coefficient (*r*) value was calculated. Data management and statistical analysis were performed with the GraphPad Prism software (San Diego, California, USA).

## 3. Results

### 3.1. Blood and Salivary Ghrelin

**Figure 1:**
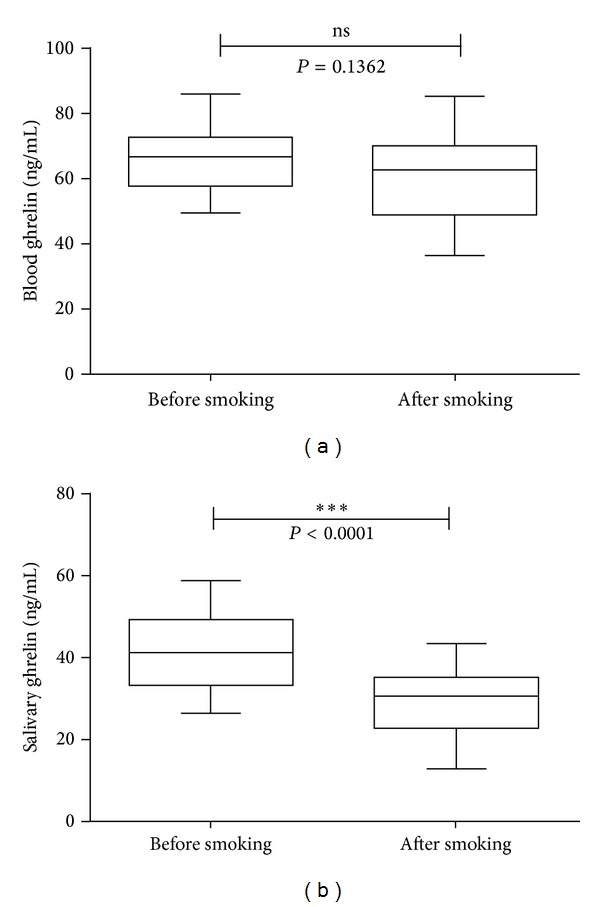
Comparison between blood and salivary ghrelin levels. Box and whiskers charts showing total ghrelin levels in blood (a) and saliva (b) before and after one-cigarette smoke. Data shown are geometric mean ± SEM of *n* = 30.

The normality test had proven that all data obtained were in fact normally distributed. The mean ± SEM values of serum total ghrelin (fasting) were 67 ± 17 ng/mL and 62 ± 24 ng/mL before and after smoking, respectively. The *t*-test comparison between the two means showed no statistical difference between the two means (*P* = 0.1362) (Figure 1). In saliva, ghrelin levels showed means of 42 ± 15 ng/mL and 28 ± 14 ng/mL before and after smoking, respectively. The latter data showed a statistically significant reduction in the ghrelin levels in saliva after smoking (*P* < 0.0001).

### 3.2. Relationship between Blood and Salivary Ghrelin

**Figure 2:**
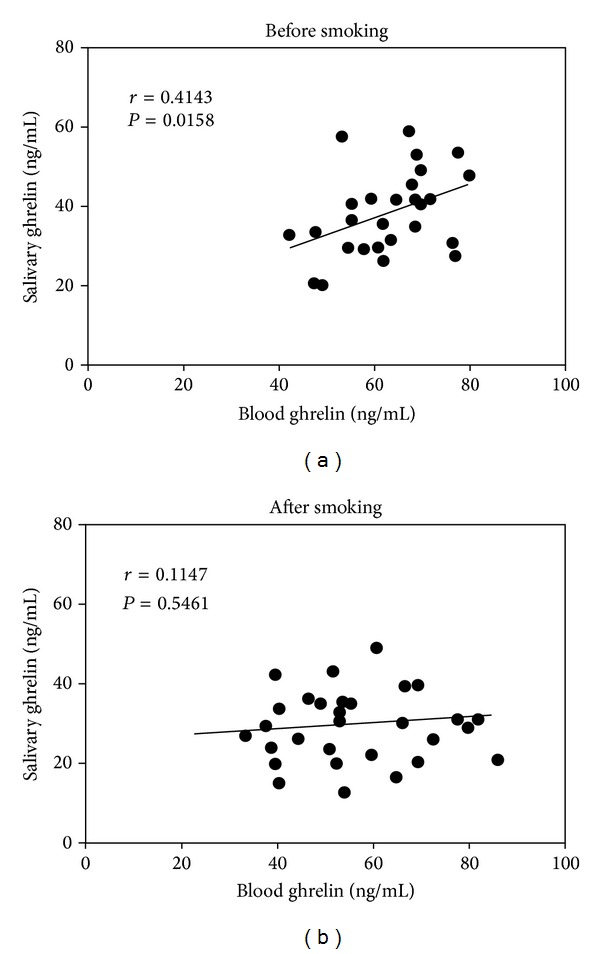
Relationship between blood and salivary ghrelin levels. Dot plots showing a positive correlation between blood and salivary ghrelin before smoking (a). However, after acute one-cigarette smoking no correlation was observed (b).

To test the association between ghrelin levels present in blood and that present in saliva, a Pearson's correlation coefficient was calculated. Blood and salivary total ghrelin values before smoking were blotted against each other and presented graphically (Figure 2). The data showed a Pearson's correlation coefficient *r* = 0.414 and *P* = 0.015, indicating a moderate positive correlation between blood and salivary total ghrelin. However, analysis of data obtained from the same subjects after one-cigarette smoking showed no statistically significant linear correlation between blood and salivary ghrelin with *r* = 0.1147 and *P* = 0.546.

## 4. Discussion

Loss of appetite, anorexia nervosa, diminished food taste, and weight loss are common in chronic cigarette smokers [2, 19]. Weight loss is probably a result of reduced calories intake and increased daily energy expenditure [12]. The appetite hormone ghrelin, in addition to other hormones, is secreted in blood to promote food intake via a very complex neuroendocrine manner [14, 15]. Ghrelin is also secreted in saliva and it has been shown to manipulate food taste; for example, it enhances sweet food taste [15] and reduces sour taste [18]. Low salivary ghrelin has been also reported in some medical conditions that might present with loss of appetite such as acute appendicitis and epileptic patients [20, 21]. These findings collectively suggested that ghrelin in saliva might contribute to maintain normal food taste and appetite. It has been shown that total ghrelin is increased in the circulation acutely after smoking [22] and decreased after smoking cessation [23]. These findings were unexpected in terms of explaining the known anorectic effect of smoking and the orexigenic effect of ghrelin. However, in both studies the salivary ghrelin has not been determined. In this self-controlled study, we have shown that acute smoking of only one cigarette can significantly reduce salivary ghrelin levels independent of those in blood. We speculated that this effect could be due to a direct impact of the nicotine on the ghrelin secretion from salivary gland or taste buds cells, a mechanism that might contribute to the dull food taste in smokers.

A lot of biomolecules and hormones found in blood are also present in saliva, and sometimes they correlate positively; that is, when their levels increase in blood they also increased in saliva and vice versa. This has suggested the use of saliva as a valuable patient sample instead of blood, as saliva sampling is easy to perform, quick, and noninvasive technique [24–26]. In line with previously reported studies [16, 17, 27], our data showed a positive correlation between blood and salivary ghrelin in healthy people. However, we reported here a direct effect of smoking on salivary ghrelin independent of that in blood. Therefore, smoking status should be considered when using saliva as a sample for ghrelin assays.

Differences between blood and salivary total ghrelin have been a controversy in different reports; some studies have shown that blood ghrelin is higher than salivary ghrelin [16] and some show the opposite [20, 27]. In this study, however, we have shown that serum ghrelin is generally higher than salivary ghrelin. These differences could be related to many factors such as health status, eating habits, regional changes, or technique used.

Although it is generally accepted now that tobacco smoking causes weight loss, the exact mechanisms are still elusive. Ghrelin and other appetite hormones are not apart from these weight changes. We have shown here that smoking acutely reduces the salivary ghrelin in healthy nonsmokers. Such effect might be even more pronounced in chronic smokers who smoke more frequently. Owing to the important role of salivary ghrelin in food taste, this effect might lead to reduced food intake. This might add another contributing factor to that responsible for weight loss in smokers. To our knowledge this is the first time such a reduction is reported; however, additional studies with higher sample size are required.

## References

[1] Shimokata H, Muller DC, Andres R (1989). Studies in the distribution of body fat. III. Effects of cigarette smoking. *Journal of the American Medical Association*.

[2] Peterson DI, Lonergan LH, Hardinge MG (1968). Smoking and taste perception. *Archives of Environmental Health*.

[3] Chiolero A, Faeh D, Paccaud F, Cornuz J (2008). Consequences of smoking for body weight, body fat distribution, and insulin resistance. *The American Journal of Clinical Nutrition*.

[4] Collins LC, Cornelius MF, Vogel RL, Walker JF, Stamford BA (1994). Effect of caffeine and/or cigarette smoking on resting energy expenditure. *International Journal of Obesity*.

[5] Hofstetter A, Schutz Y, Jequier E, Wahren J (1986). Increased 24-hour energy expenditure in cigarette smokers. *The New England Journal of Medicine*.

[6] Chen H, Vlahos R, Bozinovski S, Jones J, Anderson GP, Morris MJ (2005). Effect of short-term cigarette smoke exposure on body weight, appetite and brain neuropeptide Y in mice. *Neuropsychopharmacology*.

[7] Chen H, Hansen MJ, Jones JE (2006). Cigarette smoke exposure reprograms the hypothalamic neuropeptide Y axis to promote weight loss. *The American Journal of Respiratory and Critical Care Medicine*.

[8] Jessen A, Buemann B, Toubro S, Skovgaard IM, Astrup A (2005). The appetite-suppressant effect of nicotine is enhanced by caffeine. *Diabetes, Obesity and Metabolism*.

[9] Perkins KA, Epstein LH, Stiller RL (1991). Acute effects of nicotine on hunger and caloric intake in smokers and nonsmokers. *Psychopharmacology*.

[10] Botella-Carretero JI, Escobar-Morreale HF, Martín I (2004). Weight gain and cardiovascular risk factors during smoking cessation with bupropion or nicotine. *Hormone and Metabolic Research*.

[11] Filozof C, Fernández Pinilla MC, Fernández-Cruz A (2004). Smoking cessation and weight gain. *Obesity Reviews*.

[12] Moffatt RJ, Owens SG (1991). Cessation from cigarette smoking: changes in body weight, body composition, resting metabolism, and energy consumption. *Metabolism: Clinical and Experimental*.

[13] Kojima M, Hosoda H, Date Y, Nakazato M, Matsuo H, Kangawa K (1999). Ghrelin is a growth-hormone-releasing acylated peptide from stomach. *Nature*.

[14] Wren AM, Seal LJ, Cohen MA (2001). Ghrelin enhances appetite and increases food intake in humans. *Journal of Clinical Endocrinology and Metabolism*.

[15] Disse E, Bussier A-L, Veyrat-Durebex C (2010). Peripheral ghrelin enhances sweet taste food consumption and preference, regardless of its caloric content. *Physiology and Behavior*.

[16] Gröschl M, Topf HG, Bohlender J (2005). Identification of ghrelin in human saliva: production by the salivary glands and potential role in proliferation of oral keratinocytes. *Clinical Chemistry*.

[17] Li B-B, Chen Z-B, Li B-C (2011). Expression of ghrelin in human salivary glands and its levels in saliva and serum in Chinese obese children and adolescents. *Archives of Oral Biology*.

[18] Shin Y-K, Martin B, Kim W (2010). Ghrelin is produced in taste cells and ghrelin receptor null mice show reduced taste responsivity to salty (NaCl) and sour (citric acid) tastants. *PLoS ONE*.

[19] Anzengruber D, Klump KL, Thornton L (2006). Smoking in eating disorders. *Eating Behaviors*.

[20] Cetinkaya Z, Aydin S, Cerrahoglu YZ, Ayten R, Erman F, Aygen E (2009). Changes in appetite hormone (ghrelin) levels of saliva and serum in acute appendicitis cases before and after operation. *Appetite*.

[21] Aydin S, Dag E, Ozkan Y (2009). Nesfatin-1 and ghrelin levels in serum and saliva of epileptic patients: hormonal changes can have a major effect on seizure disorders. *Molecular and Cellular Biochemistry*.

[22] Bouros D, Tzouvelekis A, Anevlavis S (2006). Smoking acutely increases plasma ghrelin concentrations. *Clinical Chemistry*.

[23] Lee H, Joe K-H, Kim W (2006). Increased leptin and decreased ghrelin level after smoking cessation. *Neuroscience Letters*.

[24] Farnaud SJC, Kosti O, Getting SJ, Renshaw D (2010). Saliva: physiology and diagnostic potential in health and disease. *TheScientificWorldJournal*.

[25] Groschl M (2009). Current status of salivary hormone analysis. *Annales de Biologie Clinique*.

[26] Miller SM (1994). Saliva testing: a nontraditional diagnostic tool. *Clinical Laboratory Science*.

[27] Aydin S, Halifeoglu I, Ozercan IH (2005). A comparison of leptin and ghrelin levels in plasma and saliva of young healthy subjects. *Peptides*.

